# Hyperglycemic Adipocyte-Derived Exosomes Cause Oxidative Stress and Lipid Peroxidation in Brain Microvascular Endothelial Cells

**DOI:** 10.3390/cimb48090896

**Published:** 2026-09-01

**Authors:** Harshal Sawant, Bowen Sun, Yuchen Li, Cindy Zhu, Regan Meyer, Komal Sodhi, Alip Borthakur, Ji Chen Bihl

**Affiliations:** Department of Biomedical Sciences, Joan C. Edwards School of Medicine, Marshall University, Huntington, WV 25701, USA; sawantha@marshall.edu (H.S.); sunb@marshall.edu (B.S.); liyuchen8999@163.com (Y.L.); zhu36@marshall.edu (C.Z.); meyer35@marshall.edu (R.M.); sodhi@marshall.edu (K.S.); borthakur@marshall.edu (A.B.)

**Keywords:** hyperglycemia, adipocyte-derived exosomes, hypoxia-reoxygenation injury, oxidative stress, lipid peroxidation, human brain microvascular endothelial cells

## Abstract

**Background/Aims**: Type 2 diabetes mellitus (T2DM) is characterized by hyperglycemia and is a risk factor for stroke. Our previous studies demonstrated that adipocyte-derived exosomes (Ad-EXs) mediate adipose–brain communication. This study investigated the effects of hyperglycemic subcutaneous and visceral Ad-EXs on brain microvascular endothelial cell (BMEC) dysfunction during ischemic injury. **Hypothesis**: Hyperglycemic Ad-EXs exacerbate stroke injury via inducing oxidative stress and lipid peroxidation in BMECs. **Methods**: EXs were isolated from primary human subcutaneous and visceral adipocytes cultured in normal glucose (NG): NG-S-Ad-EXs and NG-V-Ad-EXs, or high-glucose (HG, 25 mM) media: HG-S-Ad-EXs and HG-V-Ad-EXs by ultracentrifugation and characterized by a nanoparticle tracking analysis system. PKH26-labeled Ad-EXs were used to evaluate uptake mechanisms in human BMECs (HBMECs). Functional effects were assessed in hypoxia/reoxygenation (H/R)-injured HBMECs treated with different Ad-EXs. Oxidative stress and lipid peroxidation markers (NOX2/4, MDA, 4-HNE, and GPX4) were analyzed. **Results**: HG increased Ad-EX release from both adipocyte depots. HG-derived Ad-EXs increased HBMEC cytotoxicity and permeability while reducing angiogenesis after H/R injury. Mechanistically, HG-Ad-EXs promoted oxidative stress through increased NOX2/4 and lipid peroxidation via elevated MDA/4-HNE and reduced GPX4 expression. **Conclusions**: Hyperglycemic Ad-EX triggered oxidative stress via NOX2/4 and lipid peroxidation via MDA/4HNE/GPX4, leading to impaired HBMEC functioning, which was exacerbated in the H/R injury condition.

## 1. Introduction

Type 2 diabetes mellitus (T2DM) is a chronic metabolic disorder primarily characterized by persistent hyperglycemia resulting from impaired insulin secretion, insulin resistance, or both. The prevalence of T2DM continues to rise worldwide as a modern-world lifestyle is increasingly being adopted [1,2]. It is predicted that the number of cases of T2DM will rise to 643 million by 2030 (International Diabetes Federation Diabetes Atlas, 9th ed.). T2DM is a major risk factor for many cerebrovascular diseases, including ischemic stroke, and contributes to worsened neurological outcomes [3]. Hyperglycemia, the hallmark metabolic abnormality of T2DM, promotes widespread cellular dysfunction, including oxidative stress, lipid dysregulation, and chronic inflammation [4]. These pathophysiological changes affect multiple organ systems and are strongly linked to the development of vascular complications.

Among the tissues affected in T2DM, adipose tissue (AT) plays a critical endocrine role. Although AT dysfunction in T2DM is well studied in the context of insulin resistance and systemic metabolic imbalance, its influence on cerebrovascular health remains poorly understood. AT is a complex organ predominantly made up of adipocytes, along with other cells collectively called the stromal vascular fraction, which includes preadipocytes, macrophages, lymphocytes, fibroblasts, endothelial cells, and stem cells. It is found that chronic hyperglycemia disrupts adipocyte homeostasis, leading to AT dysfunction marked by altered lipid handling, increased production of inflammatory secretomes, and impaired metabolic signaling [5]. AT, particularly adipocytes, are considered major producers of extracellular vesicles (EVs), particularly exosomes (EXs), the smallest homologs of EVs that exist in the size range of 10–150 nm and serve as key carriers of bioactive cargo, including lipids, proteins, and microRNAs. Adipocyte-derived exosomes (Ad-EXs) are also considered a major source of circulating EXs in the serum [6,7]. In the context of T2DM, hyperglycemia is known to alter the composition and function of Ad-EXs, making them potential mediators of systemic metabolic complications [8].

Clinical evidence suggests that T2DM markedly increases the risk of cerebrovascular disease, and people with diabetes have a higher incidence of ischemic stroke and worse outcomes after stroke compared with non-diabetic individuals [9]. Brain microvascular endothelial cells (BMECs), critical components of the blood–brain barrier and regulators of cerebral perfusion, are particularly vulnerable to hyperglycemic injury: high glucose (HG) exposure induces endothelial oxidative stress, activation of pro-oxidant pathways (e.g., NADPH oxidases/PKC), apoptosis, and barrier disruption in BMEC models [10,11,12]. Lipid peroxidation is a recurring feature of hyperglycemia-driven endothelial injury [13] and is likely a contributor to increased cerebrovascular vulnerability in diabetes [14,15]. However, the specific mechanisms through which hyperglycemic adipocytes communicate pathogenic signals to BMECs remain to be defined.

Although EXs have been implicated in inter-organ metabolic crosstalk, and EXs from AT modulate peripheral insulin sensitivity and inflammation [8,16,17], the specific role of Ad-EXs in cerebrovascular injury, particularly whether Ad-EXs under hyperglycemic conditions induce oxidative stress and lipid peroxidation in BMECs, has not been directly addressed. Related work has examined therapeutic effects of adipose-derived stem cell (ADSC)-derived EXs in models of stroke (for example, ADSC exosome-mediated pro-angiogenic signaling), but these studies do not test EXs released by mature, hyperglycemia-stressed adipocytes as pathogenic mediators of cerebrovascular injury [18]. Therefore, a mechanistic investigation of hyperglycemic Ad-EXs on BMEC redox biology would fill an important gap linking diabetic adipose dysfunction to cerebrovascular vulnerability.

AT is anatomically and functionally heterogeneous in nature. Research shows that subcutaneous adipose tissue (SAT) and visceral adipose tissue (VAT) exhibit distinct metabolic and inflammatory characteristics. VAT is more strongly associated with insulin resistance, cardiometabolic disease, and inflammatory signaling compared with SAT [19]. VAT adipocytes demonstrate elevated lipolytic activity and increased secretion of pro-inflammatory mediators, contributing to systemic metabolic dysfunction [20]. Importantly, visceral adiposity is a stronger predictor of cerebrovascular disease and ischemic stroke risk than overall adiposity, highlighting the pathogenic importance of VAT in cerebrovascular outcomes [21]. Emerging evidence suggests that these depot-specific differences extend to EX biology: VAT-derived EXs carry more pro-inflammatory and metabolically disruptive cargo than SAT-derived EXs, influencing systemic insulin sensitivity and inflammatory pathways [22]. Studying these depot-specific exosomal effects may reveal why individuals with greater visceral fat burden experience worse endothelial dysfunction and increased cerebrovascular vulnerability in T2DM.

In this study, we investigated how EXs derived from subcutaneous (S-Ad-EXs) and visceral adipocytes (V-Ad-EXs) exposed to hyperglycemic (HG) conditions impair BMEC function by induced redox imbalance and lipid peroxidation in BMECs. Our findings provide mechanistic insight into how diabetic AT may contribute to cerebrovascular injury, with implications for stroke severity in individuals with T2DM. The mechanistic insights presented in this study will help the development of future therapeutic approaches in T2DM-associated cerebrovascular complications.

## 2. Materials and Methods

### 2.1. Cell Culture

Human primary subcutaneous pre-adipocyte (ATCC, Manassas, VA, USA), human primary visceral pre-adipocyte (ScienCell, Carlsbad, CA, USA), and human brain microvascular endothelial cell (HBMECs, Cell Systems, Kirkland, WA, USA) were used for this study. Pre-adipocytes were cultured with preadipocyte medium (ATCC, Manassas, VA, USA) till 100% confluency and terminally differentiated for 7 days with preadipocyte differentiation medium (ATCC, Manassas, VA, USA) as per the company’s instructions. HBMECs were cultured in complete classic medium with serum and cultureboost (Cell Systems, Kirkland, WA, USA) and 1% penicillin/streptomycin (P/S) antibiotics in an incubator with 5% CO_2_ at 37 °C as per the manufacturer’s instructions. The culture medium was replaced every 2 days.

### 2.2. Oil Red-O Staining of Adipocytes

Oil Red-O dye (Sigma-Aldrich, St. Louis, MO, USA) was used to perform oil red-O (ORO) staining of differentiating pre-adipocytes following the company’s described protocol. In brief, after 7 days of differentiation, adipocytes were fixed for 10 min with 10% formalin at room temperature (RT). After discarding the formalin, 60% isopropanol was added for 5 min at RT. The cells were air-dried and ORO solution was added for 10 min at RT, followed by immediately washing with ddH_2_O several times. Images were taken under an inverted microscope (MAE-31R, Motic Swift Line microscope, W.E.L. Instrument Co., LLC, Mars, PA, USA) for intensity analysis. Next, 100% isopropanol was added for 10 min by shaking to elute the ORO dye. Optical density (OD) was measured at 500 nm using 100% isopropanol as a blank for further analysis.

### 2.3. Adipocyte-Derived Exosome Isolation

Pre-adipocytes (visceral and subcutaneous) were terminally differentiated for 7 days and then treated with either normal glucose (NG, 5.5 mM) or high glucose (HG, 25 mM) media for 24 h [11]. Two days before the exosome isolation, the media were changed to DMEM serum-free media with glucose (5.5 mM). Following our previously described protocol, the Ad-EXs were isolated [23,24,25,26]. In brief, the media were then collected and centrifuged at 2000 *g* for 20 min to remove cell debris. Cell culture supernatant was collected and centrifuged at 20,000 *g* for 70 min to collect microvesicles. Cell culture supernatant was then collected and ultracentrifuged using the Sorvall 120+ Micro-Ultracentrifuge (Thermo Fisher, Waltham, MA, USA) at 170,000 *g* for 90 min to collect EXs. The EXs generated from the NG- or HG-treated visceral or subcutaneous adipocytes were named NG-V-Ad-EXs, HG-V-Ad-EXs, NG-S-Ad-EXs, and HG-S-Ad-EXs, representatively.

### 2.4. Nanoparticle Tracking Analysis

The size and concentration of Ad-EXs were determined by nanoparticle tracking analysis (NTA) using NS300 (Malvern Panalytical, Amesbury, UK) as described by us before [24,27,28]. NTA visualizes and measures particle size and concentration by utilizing light scattering and Brownian motion properties. It can detect the size distribution of particles in solution from 10 nm to 2 μm in diameter. The optimum particle concentration detected by NTA is ~ 10^7^–10^9^ particles/mL. For better detection, the EX samples were diluted with sterile-filtered phosphate-buffered saline (PBS) to a concentration of 10^7^–10^8^ particles/mL. After diluting the sample, 700 μL of the same was loaded in the instrument for movement tracking at the rate of 30 frames/s. The videos with particle movement were recorded at least 3 times per sample at different positions, which were analyzed by the NTA software (version 3.5, Nanosight, Malvern Panalytical, Amesbury, UK). The NTA results were produced as a mean of the 3 tests performed per sample, and the absolute particle concentration was calculated after considering the dilution factor.

### 2.5. Hypoxia/Reoxygenation Injury

HBMECs were subjected to HG plus hypoxia/reoxygenation (H/R) to induce injury as an in vitro model of ischemic stroke in diabetes [25,29]. For inducing H/R injury, HBMECs were cultured in complete medium for 24 h (till 80% confluent) and then placed in a hypoxia chamber (1% O_2_, 5% CO_2_, and 94% N_2_, Biospherix, Parish, NY, USA) for 6 h. Following this, the HBMECs were reoxygenated for 48 h in a standard incubator (37 °C and 5% CO_2_), during which the Ad-EXs (5 × 10^8^ EX particles/mL) were added for co-incubation.

### 2.6. Incorporation of Ad-EXs via PKH-26 Labeling

To label the Ad-EXs, PKH26, a lipophilic membrane dye exhibiting red fluorescence, was used as per our previous publications [25,27,30]. The isolated Ad-EXs were incubated with 2 μL of PKH26 (2 × 10^−6^ M, Sigma-Aldrich) in 1 mL PBS for 5 min. To stop the reaction, 1 mL of 1% BSA was added and incubated for one min. The suspension was then ultracentrifuged at 170,000× *g* for 90 min to obtain the fluorescently labeled EXs. The supernatant was discarded, and the pellet was resuspended in EC medium for further co-incubation with the HBMECs.

### 2.7. Exosome Uptake Pathway Study

To determine the Ad-EX uptake mechanisms by HBMECs, we focused on the endocytic uptake pathways, including macropinocytosis, clathrin- and caveolin-dependent pathways. For this, the cells were pre-treated for 30 min with various inhibitors: 80 μM dynasore (dynamin inhibitor, Sigma-Aldrich), 200 μM genistein (caveolin inhibitor, EMD Millipore, Norwood, OH, USA), 5 μM LY290042 (macropinocytosis inhibitor, Enzo Life Sciences, Long Island, NY, USA), 10 μM pitstop 2 (clathrin inhibitor, Abcam, Cambridge, UK), or 200 μM MBCD (cyclodextrin inhibitor, Sigma-Aldrich, St. Louis, MO, USA). The concentration of the drugs was determined in accordance with our previous studies [25,27,30]. After treatment, the cells were washed twice with PBS, and then the PKH-labeled Ad-EXs were co-incubated with the HBMECs for 48 h. Fluorescent images were obtained by a fluorescence microscope (EVOS, Thermo Fisher, Waltham, MA, USA), and fluorescence intensity was quantified using ImageJ software (version 1.54, NIH, Bethesda, MD, USA).

### 2.8. Co-Incubation Experiment/Experimental Groups

For functional studies, HBMECs in either a no-injury or H/R-injury condition were co-incubated with various Ad-EXs (5 × 10^8^ particles/mL) for 48 h. There were four treatment groups: Control (HBMECs in culture medium), Vehicle (HBMECs treated with the same volume of culture medium used for EX suspension), NG-S-Ad-EXs (HBMECs treated with normal subcutaneous Ad-EXs), NG-V-Ad-EXs (HBMECs treated with normal visceral Ad-EXs), HG-S-Ad-EXs (HBMECs treated with hyperglycemic subcutaneous Ad-EXs), and HG-V-Ad-EXs (HBMECs treated with hyperglycemic visceral Ad-EXs).

### 2.9. Lactate Dehydrogenase Assay

Post 48 h of exosome treatment, HBMEC culture media were collected and used for the lactate dehydrogenase (LDH) assay kit (Invitrogen, Carlsbad, CA, USA) following the instructions provided by the manufacturer. LDH is a stable enzyme normally present inside cells. When the plasma membrane is damaged or cells undergo lysis, LDH is released into the culture medium. Thus, 490 nm provides the primary LDH signal, while 680 nm serves as a background/reference measurement. The LDH activity was measured in HBMECs by reading the absorbance at 490 nm and 680 nm, followed by calculations for the determination of percent cytotoxicity. The corrected LDH signal is commonly calculated as: corrected absorbance = A_490_ − A_680_.

### 2.10. Permeability Assay

The permeability of the HBMEC monolayer to FITC-dextran (MW, 70 k Da) was measured according to the manufacturer’s instructions provided with the vascular permeability assay kit using the Transwell model (Sigma-Aldrich, Billerica, MA, USA) [31]. In brief, the HBMEC monolayer (4 × 10^4^/cm^2^) was cultured on the Transwell inserts and treated with Ad-EXs from different groups for 48 h. FITC-dextran (1:40 dilution in phenol red-free endothelial basal medium) was added to the upper chamber, and 1 h later, 200 µL basolateral medium was aliquoted in a 96-well plate to detect FITC-dextran at fluorescence intensity (Ex: 485 nm/Em: 535 nm).

### 2.11. Tube Formation Assay

The tube formation ability of HBMECs was evaluated by using an in vitro angiogenesis assay kit (Sigma Aldrich) following the manufacturer’s protocol. Briefly, after co-culturing with different treatments, the HBMECs were trypsinized and reseeded at a density of 5 × 10^3^–1 × 10^4^ onto the surface of the polymerized ECMatrix™ and incubated at 37 °C in a CO_2_ incubator for 12–16 h. The tube formation was inspected under an inverted light microscope (MAE-31R, Motic Swift Line microscope, W.E.L. Instrument Co., LLC, Mars, PA, USA). Five independent fields were assessed for each well, and the average number of tubes per field (magnification, 40×) was determined.

### 2.12. Dihydroethidium Assay for Oxidative Stress

Intracellular reactive oxygen species (ROS) generation is assessed to determine cell oxidative stress. For dihydroethidium (DHE) staining, after 24 h of co-culture with the various types of Ad-EXs, a 10 μM DHE solution was added to the culture medium of each well of the HBMECs, and the plates were incubated at 37 °C for 70 min. Then, the medium was removed, and the cells were rinsed with PBS twice. The fluorescence signal of the cells was observed under a fluorescence microscope [25,32]. The fluorescence intensity of four randomly chosen microscopic areas in each well was analyzed using Image J (NIH, Bethesda, MD, USA). The cells cultured with special fibroblast media in a standard CO_2_ incubator were used as controls. The experiment was repeated five–six times. The images were all taken with the same gain/intensity with the same threshold (magnification, 100×). The relative fluorescence intensity was normalized to that of the control group (without hyperglycemia and hypoxia challenge). In addition, the percentage of DHE-positive cells was analyzed using the flow cytometer (BD Biosciences, San Jose, CA, USA).

### 2.13. Malondialdehyde Assay

Post 48 h of treatment, the malondialdehyde (MDA) assay (Sigma-Aldrich, cat# MAK568) was performed following the protocol provided by the manufacturer. The MDA assay is commonly used as an indicator of lipid peroxidation and oxidative stress. MDA is a reactive aldehyde generated during the oxidation of polyunsaturated fatty acids in cell membranes. In brief, 600 µL of thiobarbituric acid was mixed and incubated with 200 µL of lysed cell sample of HBMECs or standard for 1 hr at 95 °C. After the incubation, the mixture was cooled in an ice bath, and 200 µL of the RT mixture was taken into a 96-well plate, followed by absorbance readout at 532 nm, measuring the absorbance of the MDA–TBA_2_ chromogenic product.

### 2.14. Lipid Peroxidation Staining

After 48 h of treatment, lipid peroxidation fluorescent staining (Invitrogen, cat# C10445) was performed on the HBMECs by following the manufacturer’s instructions. In brief, after the treatment of HBMECs, Image-iT^®^ Lipid Peroxidation Sensor, at a concentration of 10 µM, was added to the cells for 30 min at 37 °C. After incubation, the cells were washed 3 times with PBS, and images were taken within 2 h for further analysis.

### 2.15. Western Blot Analysis

After 48 h of treatment, the proteins of HBMECs in different groups were extracted with cell lysis buffer (Fisher Scientific, Waltham, MA, USA) supplemented with a complete mini protease inhibitor tablet (Roche, Basel, Switzerland). Then, the protein lysates were electrophoresed through an SDS-PAGE gel and transferred onto PVDF membranes. The membranes were blocked with 5% non-fat milk for 1 h at room temperature and incubated with primary antibodies against NADPH oxidase 2 (NOX2, 1:1000; Abcam, Boston, MA, USA), NOX4 (1:1000; Abcam, Boston, MA, USA), MDA (1:500; Abcam, Boston, MA, USA), 4-hydroxynonenal (4HNE, 1:500; Abcam, Boston, MA, USA), glutathione peroxidase 4 (GPX4, 1:500; Sigma-Aldrich, St. Louis, MO, USA), Occludin (1:1000; Invitrogen, Carlsbad, CA, USA), Zonula Occludens-1 (ZO-1, 1:1000; Invitrogen, Carlsbad, CA, USA), Adiponectin (1:100; Invitrogen, Carlsbad, CA, USA), Fatty Acid-Binding Protein 4 (FBAP4, 1:1000; Invitrogen, Carlsbad, CA, USA), Perilipin-1 (1:1000; Invitrogen, Carlsbad, CA, USA), Peroxisome Proliferator-Activated Receptor gamma (PPAR-γ, 1:1000; Invitrogen, Carlsbad, CA, USA), TSG-101 (1:1000; Abcam, Boston, MA, USA), CD63 (1:1000; Abcam, Boston, MA, USA) and β-actin (1:4000; Sigma, St. Louis, MO, USA) at 4 °C overnight. The next day, the membranes were washed and incubated with horseradish peroxidase-conjugated anti-rabbit or anti-mouse IgG (1:40,000; Jackson Immuno Research Lab, West Grove, PA, USA) for 1.5 h at room temperature. After detection of the target protein, membranes were stripped using Restore™ PLUS Western Blot Stripping Buffer (Thermo Fisher Scientific, Waltham, MA, USA) according to the manufacturer’s instructions and re-probed with antibodies against additional target proteins or β-actin as appropriate. Consequently, the same loading control (β-actin) is shown for proteins analyzed on the same stripped and re-probed membrane. Blots were developed with enhanced chemiluminescence developing solutions, and images were quantified using ImageJ (software version 1.54, NIH, Bethesda, MD, USA).

### 2.16. Statistical Analysis

Data are expressed as the mean ± SD. Multiple comparisons were analyzed by two-way ANOVA followed by the Tukey post hoc test. GraphPad Prism 10 was used for analyzing the data. For all measurements, *p* < 0.05 was considered statistically significant.

## 3. Results

### 3.1. High-Glucose Treatment of Adipocytes Led to Increased Release of Ad-EXs

**Figure 1:**
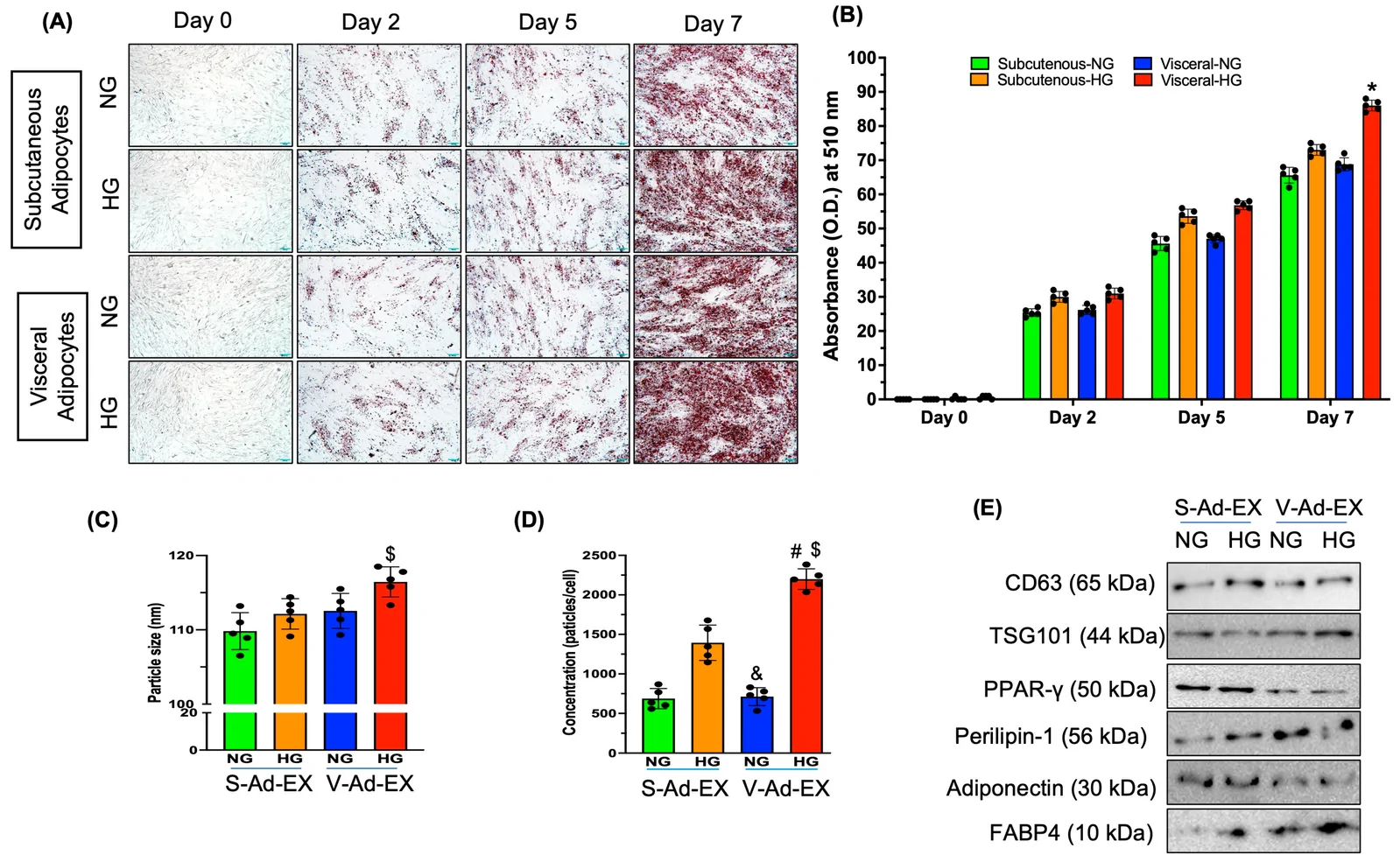
High-glucose treatment increases differentiation (maximum at day 7) and Ad-EX release of both subcutaneous and visceral adipocytes. (**A**) Representative ORO staining of differentiated subcutaneous and visceral adipocytes shows the highest number of ORO-positive cells on day 7, with higher ORO staining in HG-visceral adipocytes. Red: lipid droplets. Magnification: 100×. (**B**) Quantification of the stained lipid droplets was performed using the eluted ORO stain by measuring absorbance at 510 nm. Visceral-HG adipocytes have significantly higher ORO-positive cells as compared to subcutaneous-HG. (**C**) Particle size comparison revealed a larger particle size of HG-visceral Ad-EXs. (**D**) Concentration comparison revealed that, upon HG treatment, both subcutaneous and visceral adipocytes released significantly higher levels of Ad-EXs than their respective NG groups. Also, HG-visceral Ad-EXs are significantly higher as compared to HG-subcutaneous Ad-EXs. *n* = 5. Data expressed as mean ± SD. * *p* < 0.05, vs. subcutaneous-HG; ^$^ *p* < 0.05, vs. HG-S-Ad-EXs; ^&^ *p* < 0.05 vs. NG-S-Ad-EXs, and ^#^ *p* < 0.05 vs. NG-V-Ad-EXs. (**E**) Western blots for exosomal surface markers of Ad-EXs. HG: high glucose; NG: normal glucose; S-Ad-EXs: subcutaneous adipocyte-exosomes; V-Ad-EXs: visceral adipocyte-exosomes; ORO: oil red O.

ORO was used to confirm the differentiation of pre-adipocytes to adipocytes before the functional studies. Representative images showed that HG treatment significantly increased ORO-positive cell numbers at day 7 for both subcutaneous and visceral pre-adipocytes (Figure 1A). Data also showed that there were more ORO-positive cells in HG-treated adipocytes than in NG-treated adipocytes (*p* < 0.05; Figure 1B). This suggests that HG increases the differentiation of adipocytes, which might be linked to hypertrophy in hyperglycemia due to increased lipid accumulation and adipogenesis.

Next, the effects of HG treatment on EX released from adipocytes were analyzed using NTA to measure particle size and concentration. NTA data showed that HG-treated visceral Ad-EXs had a significantly larger size as compared to HG-treated subcutaneous Ad-EXs (*p* < 0.05; Figure 1C). More importantly, HG treatment significantly increased Ad-EX release from both subcutaneous and visceral adipocytes as compared to their NG-treatment group (*p* < 0.05; Figure 1D). HG-treatment significantly increased Ad-EX release from visceral adipocytes as compared to subcutaneous adipocytes (*p* < 0.05, vs. HG-S-Ad-EXs). Western blot analysis was performed to confirm the purity of the Ad-EXs, showing exosomal surface markers (CD63, TSG101) as well as adipocyte-specific markers (FABP4, Perilipin-1, adiponectin, PPAR- γ) (Figure 1E).

### 3.2. Hyperglycemic Ad-EXs Incorporated More into H/R-Injured HBMECs via the Micropinocytosis-Mediated EX Uptake Pathway

To determine the function of Ad-EXs on human BMECs (HBMECs), co-culture experiments were performed. First, the dose and time-dependent experiments were performed to determine the optimal time and dosage for the incorporation of Ad-EXs in HBMECs for both S-Ad-EXs and V-Ad-EXs (Supplemental Figure S1). The maximum incorporation of both PKH-26 fluorescently labeled S/V-Ad-EXs was found to be at a concentration of 5 × 10^8^ EXs/mL and a timepoint of 48 h. There were no differences in the incorporation of NG-treated S/V-Ad-EXs compared to their HG-treatment counterparts.

Secondly, the exosomal uptake pathway mechanisms were also investigated. The exosome uptake inhibitor studies showed that HBMECs uptake S/V-Ad-EXs via dynamin, caveolin, micropinocytosis, and clathrin-mediated mechanisms (*p* < 0.05, vs. vehicle) (Supplemental Figure S2). Our findings also showed that the uptake of S/V-Ad-EXs by HBMECs under H/R injury was significantly blocked by the PI3K pathway inhibitor. This observation implies that H/R injury damages the HBMEC membrane, which potentially results in an increased micropinocytosis mechanism for Ad-EX uptake.

### 3.3. Hyperglycemic Ad-EXs Caused Cytotoxicity and Impairment of the Tube Formation Ability of HBMECs

**Figure 2:**
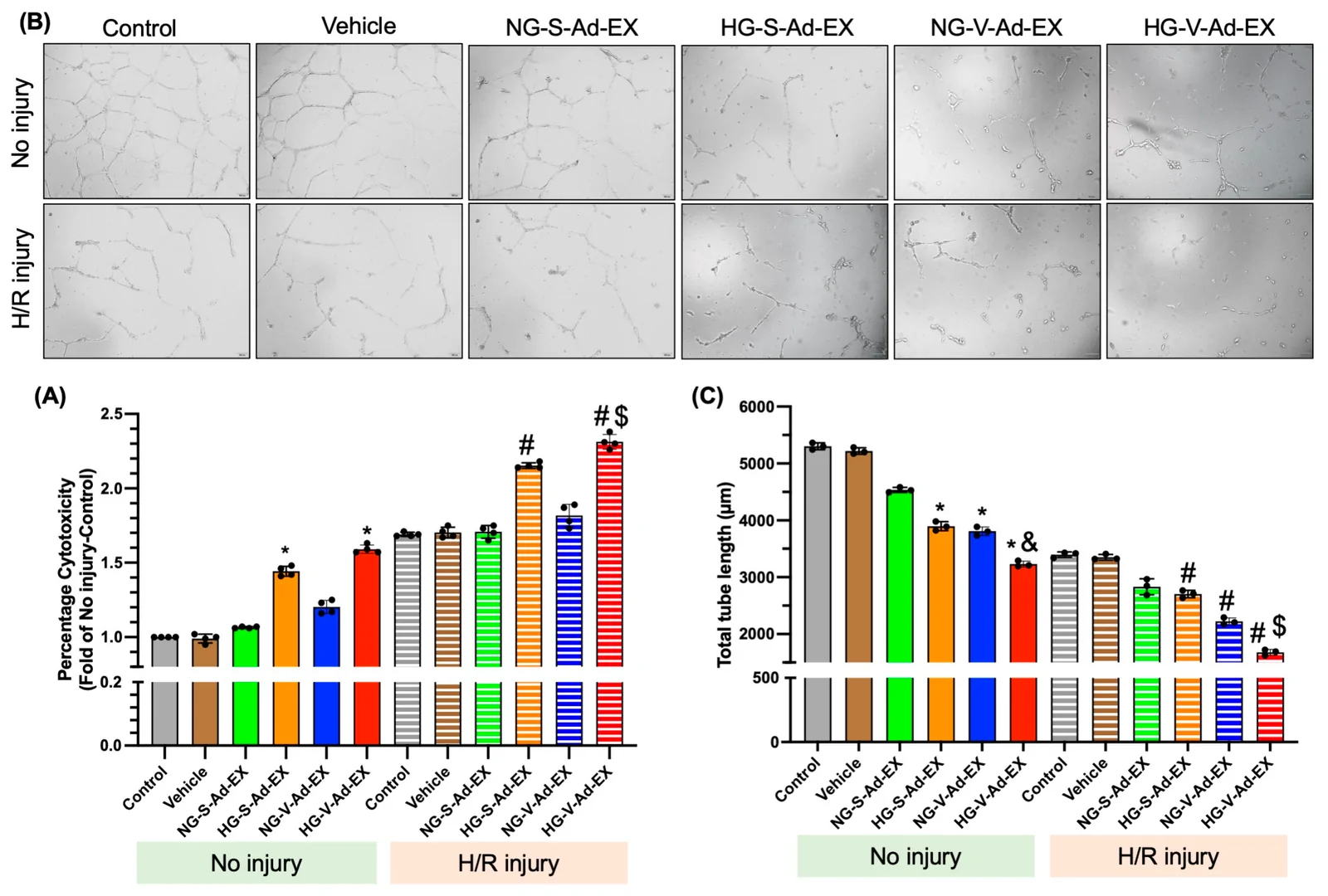
HG-treated S-Ad-EXs and V-Ad-EXs induce cytotoxicity and impair the tube formation ability in HBMECs. (**A**) Lactate dehydrogenase assay revealed increased cytotoxicity of HBMECs treated with HG-S-Ad-EXs and HG-V-Ad-EXs in both no-injury and H/R-injury conditions. (**B**) Representative images of the tube formation assay. Magnification: 40×. (**C**) Quantification of total tube length. The tube formation assay revealed decreased total length of tube formation of HBMECs treated with HG-S-Ad-EXs and HG-V-Ad-EXs in both no-injury and H/R-injury conditions. * *p* < 0.05 vs. no-injury control or NG-S/V-Ad-EXs; ^#^ *p* < 0.05 vs. H/R-injury control or NG-S/V-Ad-EXs; ^&^ *p* < 0.05 vs. no-injury-HG-S-Ad-EXs; ^$^ *p* < 0.05 vs. H/R-injury-HG-S-Ad-EXs. *n* = 5. Data expressed as mean ± SD. HG-S-Ad-EXs: HG-treated subcutaneous Ad-EXs; HG-V-Ad-EXs: HG-treated visceral Ad-EXs; H/R: hypoxia/reoxygenation; HBMECs: human brain microvascular endothelial cells.

Moreover, the function of Ad-EXs on HBMECs was determined after co-incubation at a concentration of 5 × 10^8^ EXs/mL for 48 h. First, the LDH assay revealed that both hyperglycemic S-Ad-EXs and V-Ad-EXs caused a significant increase in cytotoxicity in both no-injury and H/R-injury conditions (*p* < 0.05, vs. NG-S/V-Ad-EXs; Figure 2A). During H/R injury, a significant increase in cytotoxicity of HBMECs was observed (*p* < 0.05, vs. no-injury). When H/R-injured HBMECs were treated with hyperglycemic S/V-Ad-EXs, there was a further increase in the total cytotoxicity level (*p* < 0.05). These findings suggest that hyperglycemic S/V-Ad-EXs induce cytotoxicity in HBMECs.

Functional assessment of HBMECs post-treatment through the tube formation assay revealed that both hyperglycemic S-Ad-EX and V-Ad-EXs significantly decreased the tube length and number of tubes of HBMECs in both no-injury and H/R-injury conditions (*p* < 0.05, vs. NG-S/V-Ad-EXs; Figure 2B,C). H/R injury significantly decreased both the number and length of tubes formed in HBMECs (*p* < 0.05, vs. no-injury). Treatment of H/R-injured HBMECs with hyperglycemic S/V-Ad-EXs resulted in a further decrease in tube formation function compared with H/R injury alone (*p* < 0.05). This suggests that hyperglycemic S/V-Ad-EXs decrease the angiogenesis ability of HBMECs.

### 3.4. Hyperglycemic Ad-EXs Caused Increased Permeability and Decreased Tight Junction Expression in HBMECs

**Figure 3:**
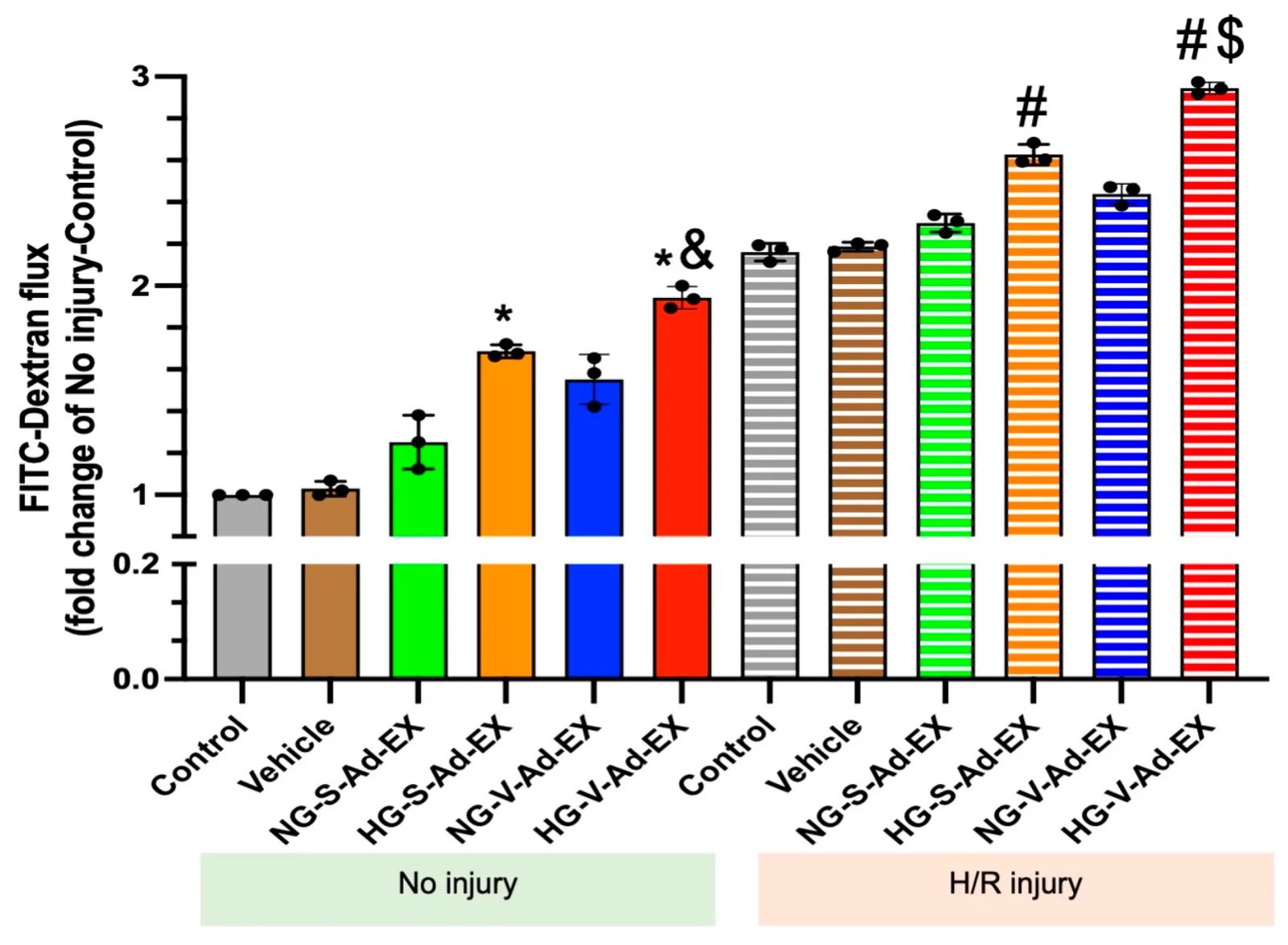
Co-culture of subcutaneous and visceral adipocytes with HBMECs revealed increased permeability of HBMECs when treated with HG-treated both subcutaneous and visceral Ad-EXs. The permeabilization assay revealed increased permeabilization of HG-S-Ad-EXs and HG-V-Ad-EXs. * *p* < 0.05 vs. no-injury control or NG-S/V-Ad-EXs; ^#^ *p* < 0.05 vs. H/R-injury control or NG-S/V-Ad-EXs; ^&^ *p* < 0.05 vs. no-injury-HG-S-Ad-EXs; ^$^ *p* < 0.05 vs. H/R-injury-HG-S-Ad-EXs. *n* = 5. Data expressed as mean ± SD. HG-S-Ad-EXs: HG-treated subcutaneous Ad-EXs; HG-V-Ad-EXs: HG-treated visceral Ad-EXs; HBMECs: human brain microvascular endothelial cells.

The permeability assay revealed that co-culturing HBMECs with hyperglycemic subcutaneous or visceral adipocytes in a Transwell caused a significant increase in the permeability of FITC-dextran through HBMECs in both no-injury and H/R-injury conditions (*p* < 0.05, vs. NG-S/V-Ad-EXs; Figure 3). This suggests that hyperglycemic S/V-Ad-EXs cause damage to the barrier function of HBMECs, which causes increased permeability.

**Figure 4:**
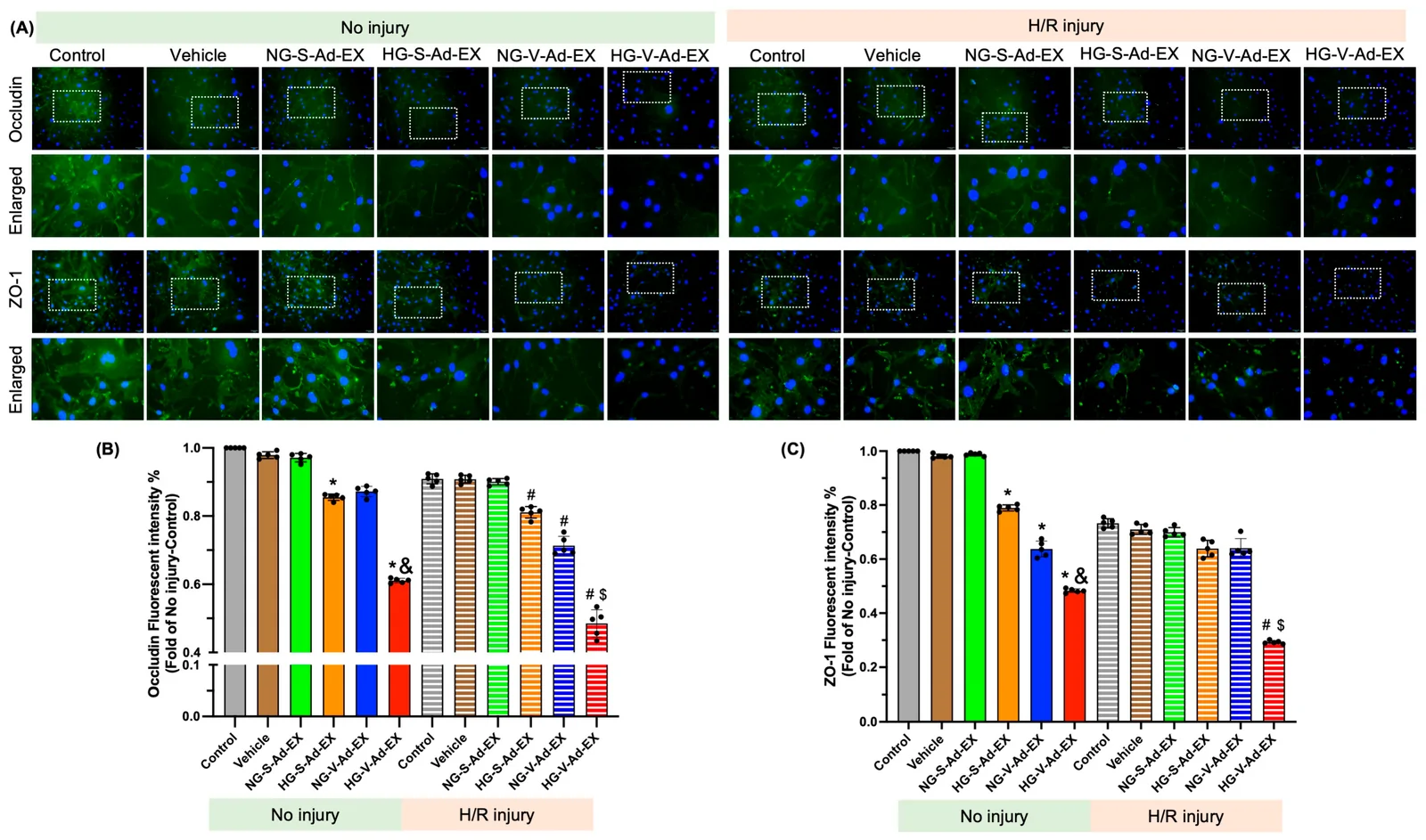
HG-S-Ad-EXs and HG-V-Ad-EXs decrease occludin and ZO-1 expression in HBMECs. (**A**) Representative images of tight junction (occludin and ZO-1) expression. Green: occludin/ZO-1, Blue: DAPI. Scale bars: 50 mm. (**B**) Quantification of Occludin expression. (**C**) Quantification of ZO-1 expression. Immunostaining revealed decreased expression of occludin and ZO-1 expression in HBMECs treated with HG-S-Ad-EXs and HG-V-Ad-EXs in both no-injury and H/R-injury conditions. * *p* < 0.05 vs. no-injury control or NG-S/V-Ad-EXs; ^#^ *p* < 0.05 vs. H/R-injury control or NG-S/V-Ad-EXs; ^&^ *p* < 0.05 vs. no-injury-HG-S-Ad-EXs; ^$^ *p* < 0.05 vs. H/R-injury-HG-S-Ad-EXs. *n* = 5. Data expressed as mean ± SD. HG-S-Ad-EXs: HG-treated subcutaneous Ad-EXs; HG-V-Ad-EXs: HG-treated visceral Ad-EXs; HBMECs: human brain microvascular endothelial cells; ZO-1: Zonula Occludens-1.

**Figure 5:**
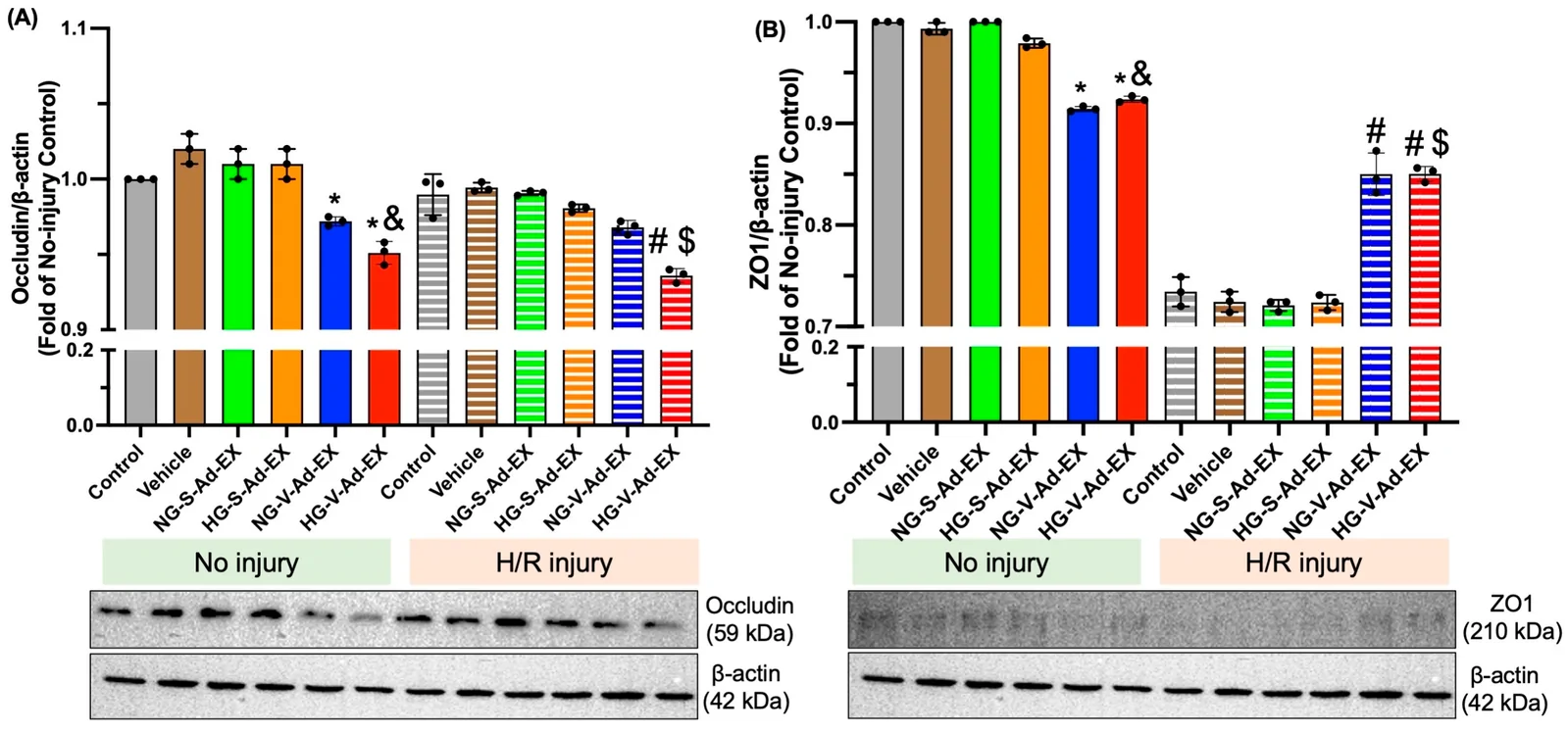
HG-S-Ad-EXs and HG-V-Ad-EXs decrease occludin and ZO-1 protein expression in HBMECs. Representative Western blot blots and quantification of occludin expression (**A**) and ZO-1 expression (**B**). The same β-actin loading control is shown because the membrane was stripped and re-probed for occludin and ZO-1 proteins. Protein assays revealed decreased expression of occludin and ZO-1 in HBMECs treated with HG-S-Ad-EXs and HG-V-Ad-EXs in both no-injury and H/R-injury conditions. * *p* < 0.05 vs. no-injury control or NG-S/V-Ad-EXs; ^#^ *p* < 0.05 vs. H/R-injury control or NG-S/V-Ad-EXs; ^&^ *p* < 0.05 vs. no-injury-HG-S-Ad-EXs; ^$^ *p* < 0.05 vs. H/R-injury-HG-S-Ad-EXs. *n* = 3. Data expressed as mean ± SD. HG-S-Ad-EXs: HG-treated subcutaneous Ad-EXs; HG-V-Ad-EXs: HG-treated visceral Ad-EXs; HBMECs: human brain microvascular endothelial cells; ZO-1: Zonula Occludens-1.

Immunofluorescent staining (Figure 4) and Western blot analysis (Figure 5) of tight junction proteins, occludin and ZO-1, showed that both hyperglycemic S-Ad-EXs and V-Ad-EXs significantly decreased their expressions in HBMECs in both no-injury and H/R-injury conditions (*p* < 0.05, vs. NG-S/V-Ad-EXs). This suggests that hyperglycemic S/V-Ad-EXs decrease the tight junctions of HBMECs, which is related to the increased permeability.

### 3.5. Hyperglycemic Ad-EXs Cause Oxidative Stress in HBMECs

**Figure 6:**
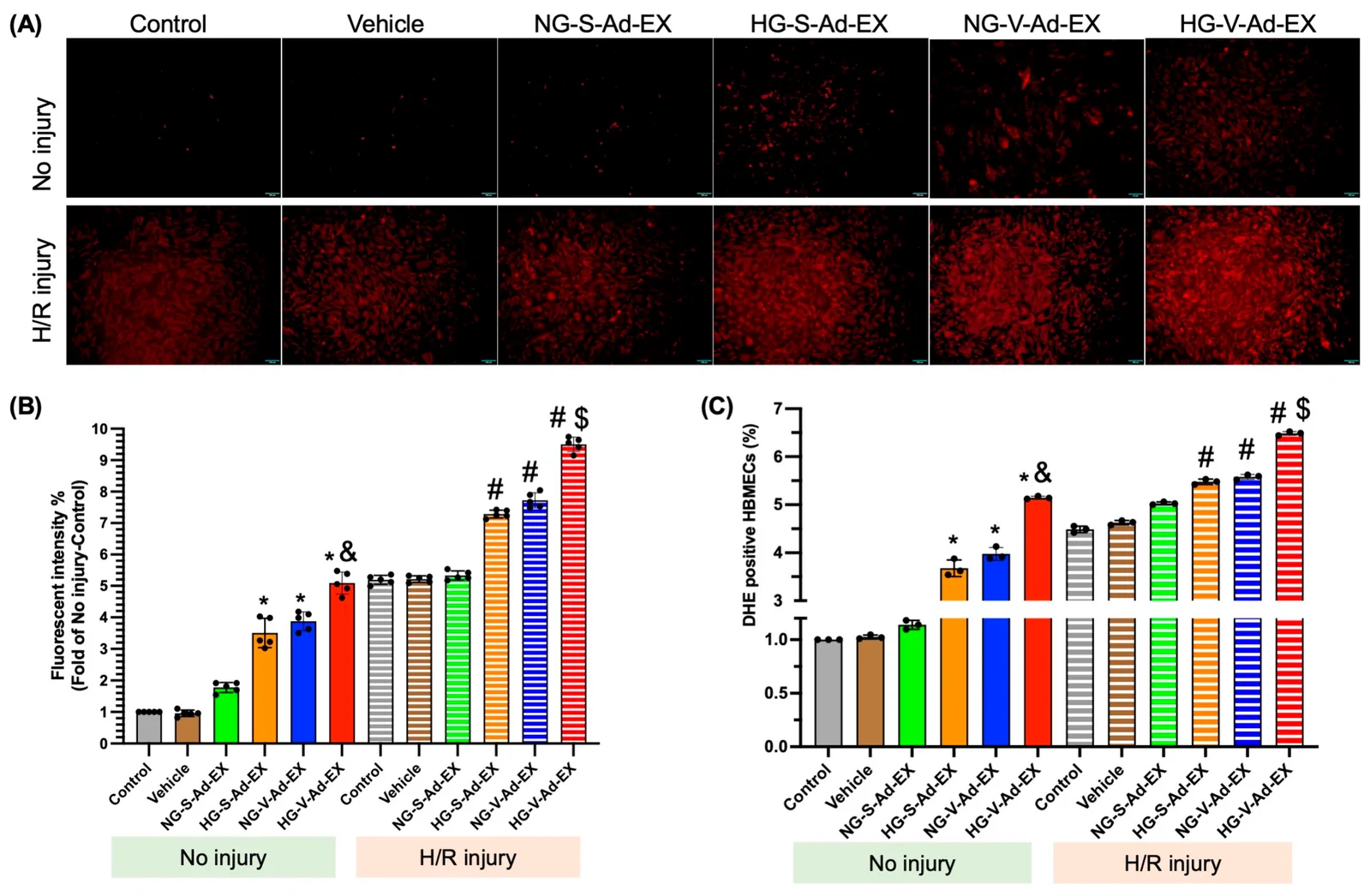
HG-S-Ad-EXs and HG-V-Ad-EXs increase the oxidative stress in HBMECs. (**A**) Representative images of DHE-staining. Red: DHE-positive HBMECs. Scale bars: 100 mm. (**B**) Quantification of fluorescence intensity using ImageJ. (**C**) Quantification by flow cytometry. DHE revealed increased oxidative stress of HBMECs treated with HG-S-Ad-EXs and HG-V-Ad-EXs in both no-injury and H/R-injury conditions. * *p* < 0.05 vs. no-injury control or NG-S/V-Ad-EXs; ^#^ *p* < 0.05 vs. H/R-injury control or NG-S/V-Ad-EXs; ^&^ *p* < 0.05 vs. no-injury-HG-S-Ad-EXs; ^$^ *p* < 0.05 vs. H/R-injury-HG-S-Ad-EXs. *n* = 5. Data expressed as mean ± SD. HG-S-Ad-EXs: HG-treated subcutaneous Ad-EXs; HG-V-Ad-EXs: HG-treated visceral Ad-EXs; DHE: dihydroethidium; HBMECs: human brain microvascular endothelial cells.

Immunofluorescent staining for ROS via DHE staining (Figure 6A,B) showed that both HG-S/V-Ad-EXs significantly increase DHE staining in HBMECs in both no-injury and H/R-injury conditions as compared to NG-S/V-Ad-EXs (*p* < 0.05). H/R injury induced a significant increase in ROS production in HBMECs when compared to the no-injury group (*p* < 0.05). Flow cytometry was also used to quantify the number of DHE-positive HBMECs and further confirmed similar findings to the immunofluorescent data (Figure 6C). The representative flow plots are shown in Supplemental Figure S3.

**Figure 7:**
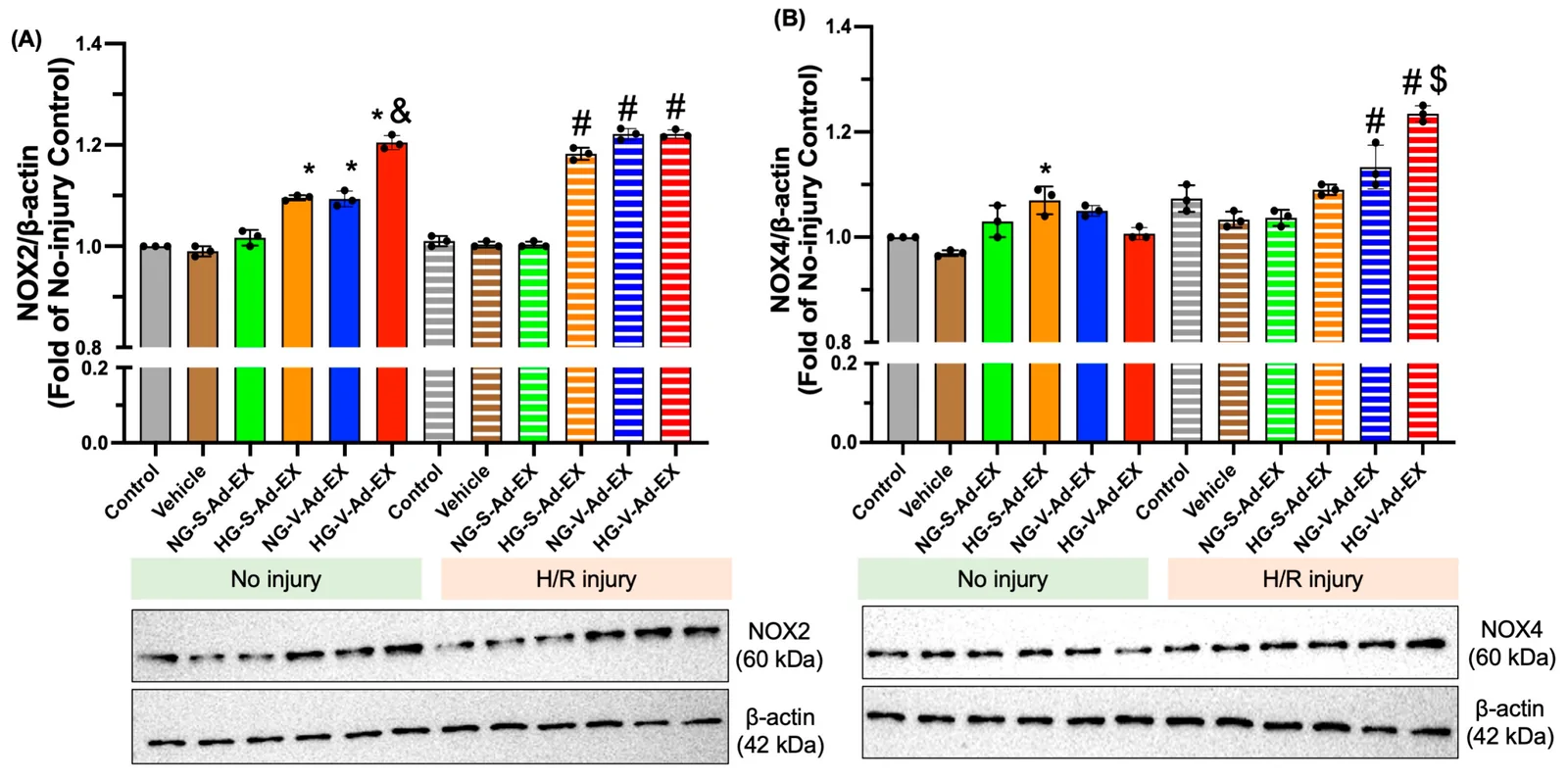
HG-S-Ad-EXs and HG-V-Ad-EXs increase NOXNOX2/4 protein expression in HBMECs. (**A**,**B**) Representative Western blot images and quantification of NOX2/4 expression. Western blot revealed increased NOX2/4 expression of HBMECs treated with HG-S-Ad-EXs and HG-V-Ad-EXs in both no-injury and H/R-injury conditions. * *p* < 0.05 vs. no-injury control or NG-S/V-Ad-EXs; ^#^ *p* < 0.05 vs. H/R-injury control or NG-S/V-Ad-EXs; ^&^ *p* < 0.05 vs. no-injury-HG-S-Ad-EXs; ^$^ *p* < 0.05 vs. H/R-injury-HG-S-Ad-EXs. *n* = 3. Data expressed as mean ± SD. HG-S-Ad-EXs: HG-treated subcutaneous Ad-EXs; HG-V-Ad-EXs: HG-treated visceral Ad-EXs; NOX: NADPH oxidase; HBMECs: human brain microvascular endothelial cells.

Western blots were used to measure the oxidative markers (NOX2/4) in the HBMECs after coincubation. Data showed that both HG-S-Ad-EXs and V-Ad-EXs caused an upregulation in NOX2/4 protein levels in HBMEC (*p* < 0.05, vs. NG-S/V-Ad-EXs; Figure 7). This suggests that hyperglycemic S/V-Ad-EXs cause oxidative stress in HBMECs via upregulating NOX2/4 expression.

### 3.6. Hyperglycemic Ad-EXs Cause Lipid Peroxidation in HBMECs

**Figure 8:**
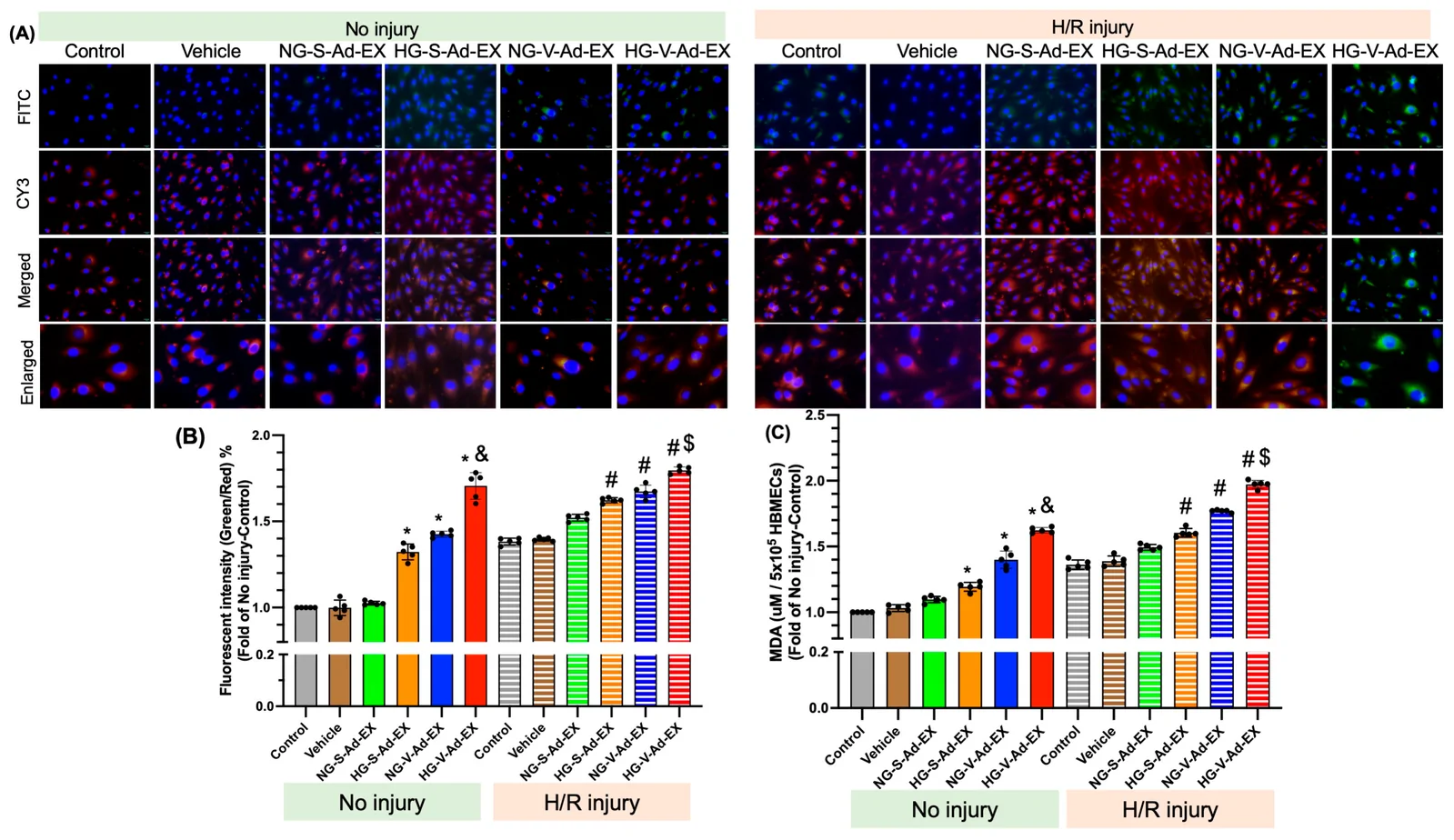
HG-S-Ad-EXs and HG-V-Ad-EXs increase lipid peroxidation in HBMECs. (**A**) Representative images of lipid peroxidation staining. Magnification: 200×. (**B**) Quantification of fluorescent intensity. (**C**) MDA assay. Lipid peroxidation staining and MDA assay revealed increased lipid peroxidation and MDA production of HBMECs treated with HG-S-Ad-EXs and HG-V-Ad-EXs in both no-injury and H/R-injury conditions. * *p* < 0.05 vs. no-injury control or NG-S/V-Ad-EXs; ^#^ *p* < 0.05 vs. H/R-injury control or NG-S/V-Ad-EXs; ^&^ *p* < 0.05 vs. no-injury-HG-S-Ad-EXs; ^$^ *p* < 0.05 vs. H/R-injury-HG-S-Ad-EXs. *n* = 5. Data expressed as mean ± SD. HG-S-Ad-EXs: HG-treated subcutaneous Ad-EXs; HG-V-Ad-EXs: HG-treated visceral Ad-EXs; MDA: malondialdehyde, a lipid peroxidation marker; HBMECs: human brain microvascular endothelial cells.

Immunofluorescent staining for lipid peroxidation revealed that both hyperglycemic S-Ad-EXs and V-Ad-EXs significantly increase lipid peroxidation in HBMECs in both no-injury and H/R-injury conditions (*p* < 0.05, vs. NG-S/V-Ad-EXs; Figure 8A,B).

Moreover, the MDA assay was performed to reaffirm the effects of HG-Ad-EXs on lipid peroxidation. The data showed that both hyperglycemic S-Ad-EXs and V-Ad-EXs significantly increased the production of MDA in HBMECs in both no-injury and H/R-injury conditions (*p* < 0.05, vs. NG-S/V-Ad-EXs; Figure 8C).

**Figure 9:**
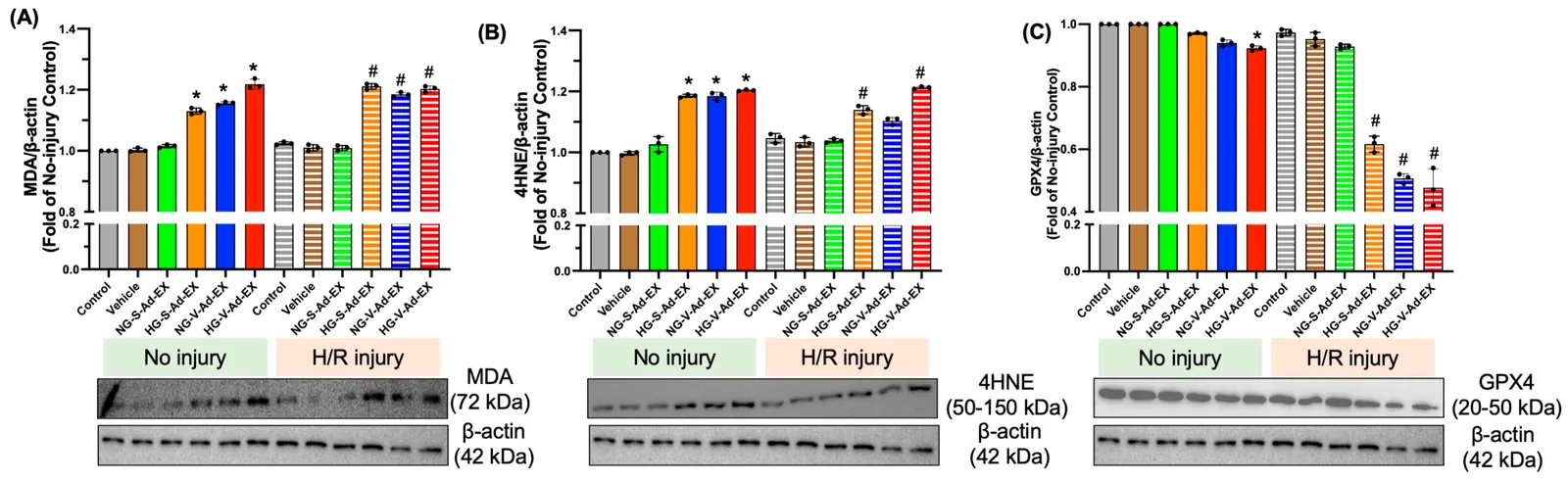
HG-S-Ad-EXs and HG-V-Ad-EXs increase protein expression of lipid peroxidation in HBMECs. Representative Western blot images and quantification of MDA (**A**), 4HNE, (**B**) and GPX4 expression (**C**). The same β-actin loading control is shown because the membrane was stripped and re-probed for MDA, 4HNE and GPX4 proteins. * *p* < 0.05 vs. no-injury control or NG-S/V-Ad-EXs; ^#^ *p* < 0.05 vs. H/R-injury control or NG-S/V-Ad-EXs. *n* = 3. Data expressed as mean ± SD. HG-S-Ad-EXs: HG-treated subcutaneous Ad-EXs; HG-V-Ad-EXs: HG-treated visceral Ad-EXs; MDA: malondialdehyde; 4HNE: 4-hydroxynonenal, a major cytotoxic end-product of lipid peroxidation; GPX4: Glutathione peroxidase 4, an antioxidant enzyme that protects cells against lipid peroxidation; HBMECs: human brain microvascular endothelial cells.

The Western blot was used to measure the protein levels of lipid peroxidative markers, MDA, 4HNE, and GPX4. Both hyperglycemic S-Ad-EXs and V-Ad-EXs increased MDA protein levels. Interestingly, normal V-Ad-EXs also increased MDA expression. A similar pattern was observed for 4-HNE under the no-injury condition; however, under the H/R injury condition, normal V-Ad-EXs did not significantly increase 4-HNE expression. In contrast, GPX4 expression was significantly decreased only by hyperglycemic V-Ad-EXs under the no-injury condition. Following H/R injury, however, both hyperglycemic S-Ad-EXs and V-Ad-EXs, as well as normal V-Ad-EXs, significantly decreased GPX4 protein levels in HBMECs (*p* < 0.05, vs. NG-S/V-Ad-EXs; Figure 9). This suggests that hyperglycemic subcutaneous and visceral Ad-EXs cause lipid peroxidation in HBMECs via regulating MDA, 4HNE, and GPX4 expressions.

## 4. Discussion

In this study, we demonstrate that hyperglycemia alters Ad-EX biogenesis and function, resulting in HBMEC dysfunction in an in vitro H/R model. These findings extend emerging evidence that Ad-EXs modulate distant vascular targets and contribute to metabolic vascular disease. Adipocyte dysfunction in the context of T2DM and obesity has been increasingly recognized not only as a source of systemic inflammation but also as a driver of endothelial pathology through adipokine and exosomal signaling. Previous research showed correlations of altered Ad-EXs cargo (pro-inflammatory proteins and microRNAs) with impaired endothelial nitric oxide bioavailability and increased oxidative stress and inflammation in diabetes [33,34], which could lead to endothelial dysfunction and vascular complications. However, whether and how Ad-EXs cause brain endothelial dysfunction linked to the cerebrovascular complications has not yet been studied. In our in vitro model, we first studied the effect of hyperglycemic Ad-EXs on unexplored BMECs for their critical role in preserving blood–brain barrier integrity and their angiogenic potential, both of which are fundamental to post-stroke vascular remodeling and neurological recovery.

Hyperglycemia and insulin resistance are well-established mediators of endothelial oxidative stress, largely through activation of the NADPH oxidase (NOX) enzyme family. Among these, NOX2 and NOX4 are particularly implicated in diabetic vascular pathology. Chronic hyperglycemia enhances their transcriptional and post-translational activation, resulting in sustained production of superoxide and hydrogen peroxide. Excessive ROS generation disrupts redox homeostasis, promotes oxidative damage to lipids and proteins, and induces eNOS uncoupling, thereby reducing nitric oxide (NO) bioavailability and impairing vasodilatory function [35]. In our model, HBMECs treated with hyperglycemic Ad-EXs exhibited upregulation of NOX2 and NOX4 expression, consistent with a pro-oxidative phenotype. This was accompanied by increased lipid peroxidation markers (MDA, 4-HNE) and reduced GPX4, an essential antioxidant enzyme that protects against lipid peroxidation and ferroptotic cell death [36]. This profile mirrors pathophysiologic mechanisms described in diabetes-associated vascular complications, where elevated ROS and diminished antioxidant defenses contribute to endothelial barrier disruption and impaired angiogenic responses [37].

Our mechanistic findings support a model in which hyperglycemic Ad-EXs act as vectors for pathological signals that amplify oxidative stress in endothelial cells. Exosomal transfer of NOX2/4 or NOX-activating signals from dysfunctional adipocytes has been implicated in other tissues; for example, adipocyte-derived exosomal NOX4 induces oxidative damage and cellular senescence in placental cells in maternal obesity [38]. We found that Ad-EXs in hyperglycemic conditions are responsible for NOX induction leading to oxidative stress in HBMECs. Moreover, Ad-EXs induced altered levels of MDA and GPX4 in HBMECs, which may cause a lipid peroxidation state. We acknowledge that the Ad-EX might carry cargo candidate mediators, including miRNAs and other proteins known to regulate redox and angiogenic pathways. Prior work has demonstrated that adipose tissue stem cell-EV cargo of miR-126 reduction in obesity can suppress eNOS activation and impede pro-angiogenic signaling [12,23,39], suggesting that similar mechanisms could underlie impaired tube formation in HBMECs. Future proteomic and transcriptomic profiling of hyperglycemic Ad-EXs will be essential to identify the specific exosomal components that are responsible for these effects and to define the underlying molecular mechanisms linking adipocyte-derived exosomes to diabetic vascular dysfunction.

The downregulation of GPX4 observed in hyperglycemic Ad-EX as well as the treatment of HMBECs further highlights a link between metabolic stress and dysregulated lipid handling in endothelial cells. GPX4 functions as a key regulator of lipid peroxidation and ferroptosis, and its depletion sensitizes cells to oxidative injury. Although GPX4’s role in diabetic vascular disease has not been extensively studied, its central position in controlling redox balance suggests that reduced GPX4 could exacerbate endothelial injury under hyperglycemia. Our data thus align with broader evidence implicating disrupted antioxidant responses as a critical mediator of vascular pathology in T2DM [37].

Clinically, endothelial dysfunction is a recognized precursor of cerebrovascular and cardiovascular disease in individuals with T2DM and hyperglycemia. NADPH oxidase-driven ROS generation and consequent NO bioavailability loss occur early in the pathogenesis of diabetic vascular complications, preceding overt clinical manifestations [35]. Our findings position hyperglycemic Ad-EXs as novel contributors to this process, potentially linking adipose metabolic stress with cerebrovascular vulnerability in ischemic stroke. This paradigm complements existing therapeutic strategies targeting oxidative stress and metabolic inflammation but suggests that modulation of exosome production or uptake might represent an upstream intervention point.

A notable finding in our study was the differential impact of hyperglycemic visceral-derived Ad-EXs compared with hyperglycemic subcutaneous-derived Ad-EXs on HBMEC injury, with visceral Ad-EXs exerting more pronounced detrimental effects across multiple endpoints. These results are consistent with established depot-specific differences in adipose tissue biology: VAT is metabolically more active, exhibits higher basal lipolytic rates, and is more closely linked with insulin resistance and systemic metabolic dysfunction than SAT. VAT expansion correlates more strongly with oxidative stress, inflammation, and cardiometabolic risk than SAT, reflecting intrinsic biological differences in cellular stress responses and secretory profiles between depots [40,41]. Emerging evidence further suggests that extracellular vesicles released from VAT depots carry distinct molecular cargo that is more pro-inflammatory and pro-metabolic dysregulation-associated than those from subcutaneous depots. In models of metabolic disease, vesicles from visceral adipose tissue induce greater pathogenic responses, such as increased inflammation, lipid dysregulation, and impaired insulin signaling, compared with SAT-derived EVs [42,43]. Our observation that hyperglycemic visceral Ad-EXs lead to higher endothelial cytotoxicity, permeability, and angiogenic impairment aligns with this depot-specific functional divergence, suggesting that visceral depots may generate exosome populations with exacerbated pathogenic cargo under hyperglycemic stress. These findings underscore the importance of adipose depot origin in shaping exosome-mediated interorgan communication and vascular injury in the context of T2DM and ischemic stroke. Importantly, the detrimental effects of hyperglycemic Ad-EXs were further magnified under H/R conditions, which model ischemia-reperfusion injury. H/R itself is known to induce ROS overproduction and disruption of endothelial tight junctions. When combined with hyperglycemic visceral-Ad-EX exposure, these stressors appeared to act synergistically, leading to heightened oxidative burden, increased lipid peroxidation, and more severe barrier dysfunction compared to either insult alone. This suggests a “two-hit” paradigm in which metabolic stress primes BMECs toward redox vulnerability, and subsequent ischemic stress exacerbates membrane damage. In the context of T2DM-associated stroke, such synergistic injury may significantly impair vascular repair processes, reduce angiogenic recovery, and prolong blood–brain barrier disruption.

In summary, our study provides mechanistic insight into how hyperglycemic Ad-EXs impair cerebral microvascular endothelial function through oxidative stress and lipid peroxidation. These data add to a growing body of evidence that adipose-endothelial exosomal signaling contributes to vascular complications in metabolic disease. All our findings are based on in vitro studies using human primary cell lines. Further validation via in vivo studies would solidify our findings. Unraveling the specific exosomal cargo and uptake pathways responsible for these effects will be crucial for the development of targeted therapies aimed at mitigating stroke risk in patients with T2DM.

## Data Availability

The raw data supporting the conclusions of this article will be made available by the authors on request.

## Supplementary Materials

The following supporting information can be downloaded at: https://www.mdpi.com/article/10.3390/cimb48090896/s1.
