# Supplementary material for: Hyperglycemic Adipocyte-Derived Exosomes Cause Oxidative Stress and Lipid Peroxidation in Brain Microvascular Endothelial Cells
Source: Curr Issues Mol Biol. 2026 Sep 1;48(9):896. doi: 10.3390/cimb48090896 (PMC13605305; doi:10.3390/cimb48090896)
Supplement: Supplementary file 1 [file cimb-48-00896-s001.zip › cimb-4452218-supplementary - Copy.pdf]

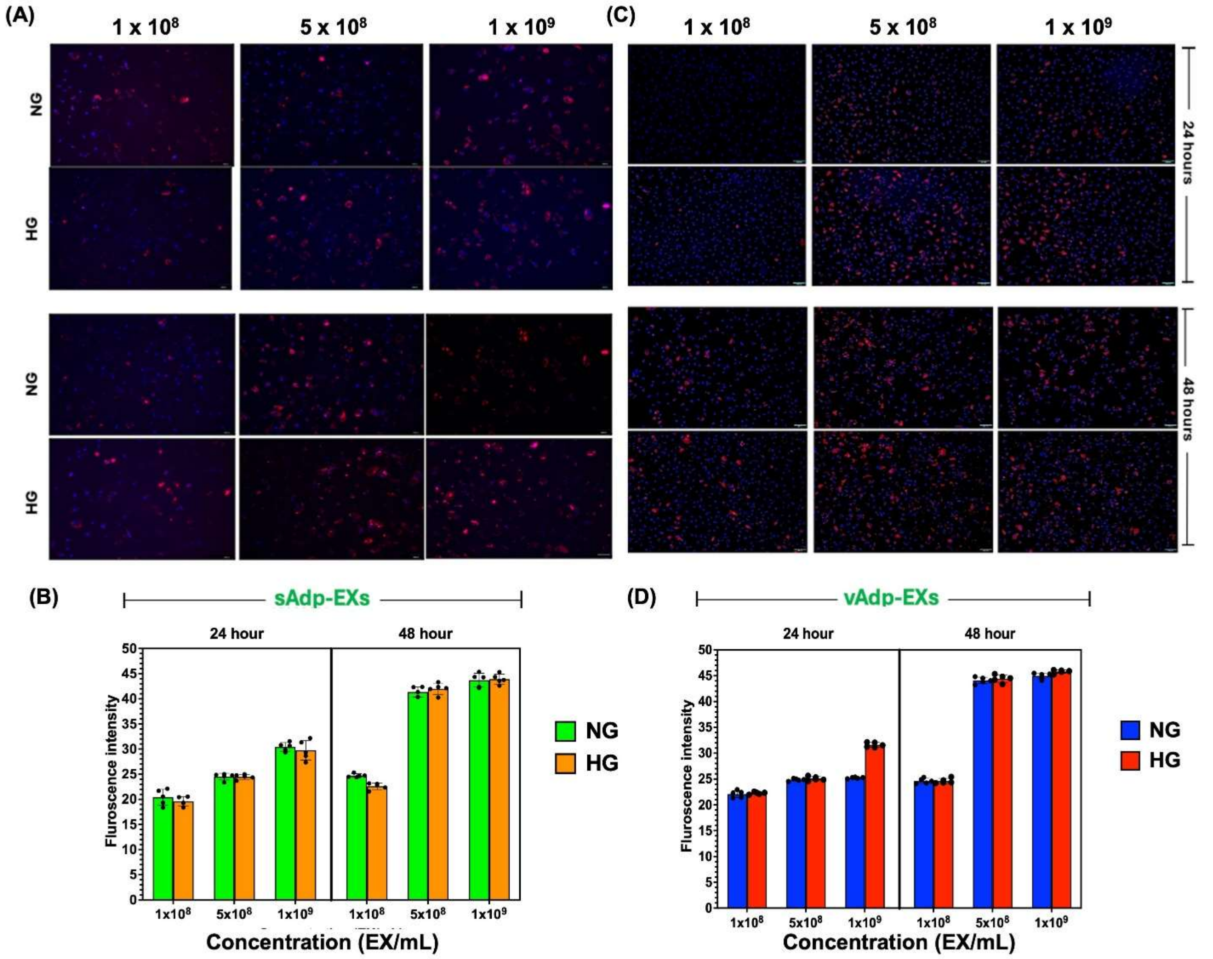

Supplemental Figure S1: Maximum incorporation of PKH-stained Ad-EXs in HBMECs is at 5 x 10^8 EXs/mL concentration and at 48 hrs of timepoint. (A) Representative images of PKH26-stained subcutaneous Ad-EXs incorporation in HBMECs. (B) Quantification of fluorescent intensity of PKH-26-stained subcutaneous Ad-EXs revealed maximum incorporation in HBMECs at 5 x 10^8 EXs/mL and at 48 hrs. (C) Representative images of PKH-26-stained visceral Ad-EXs incorporation in HBMECs. (D) Quantification of fluorescent intensity of PKH-26-stained visceral Ad-EXs revealed maximum incorporation in HBMECs at 5 x 10^8 EXs/mL and at 48 hrs. No significant differences were observed in the incorporation of subcutaneous and visceral Ad-EXs in HBMECs at 48 hours. Red: PKH-26-stained Ad-EXs, Blue: DAPI-nuclei of HBMECs. Scale Bars: 100 µm. Magnification: 100X.

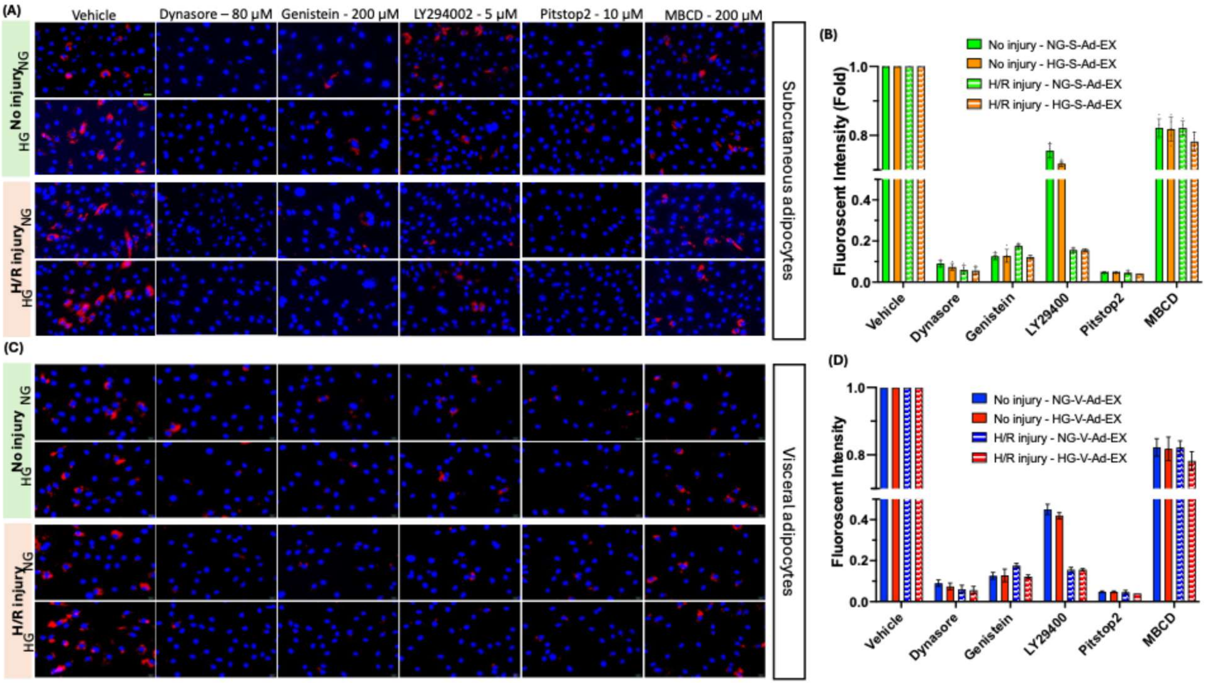

Supplemental Figure S2: Co-incubation of PKH-26-stained Ad-EXs with HBMECs in the presence of exosome uptake inhibitors. (A) Representative images of PKH26-stained subcutaneous Ad-EXs incorporation in HBMECs in the presence of exosome uptake inhibitors. (B) Quantification of fluorescent intensity of PKH-26-stained subcutaneous Ad-EXs revealed that dynamin, genistein and pitstop 2 significantly decrease the exosome uptake. (C) Representative images of PKH-26-stained visceral Ad-EXs incorporation in HBMECs in the presence of exosome uptake inhibitors. (D) Quantification of fluorescent intensity of PKH-26-stained visceral Ad-EXs revealed that dynamin, genistein and pitstop 2 significantly decrease the exosome uptake. There was a significant decrease in visceral Ad-EXs uptake in H/R-injured HBMECs in the presence of LY29400. Red: PKH-26-stained Ad-EXs, Blue: DAPI-nuclei of HBMECs. Scale Bars: 50 μm. Magnification: 180X.

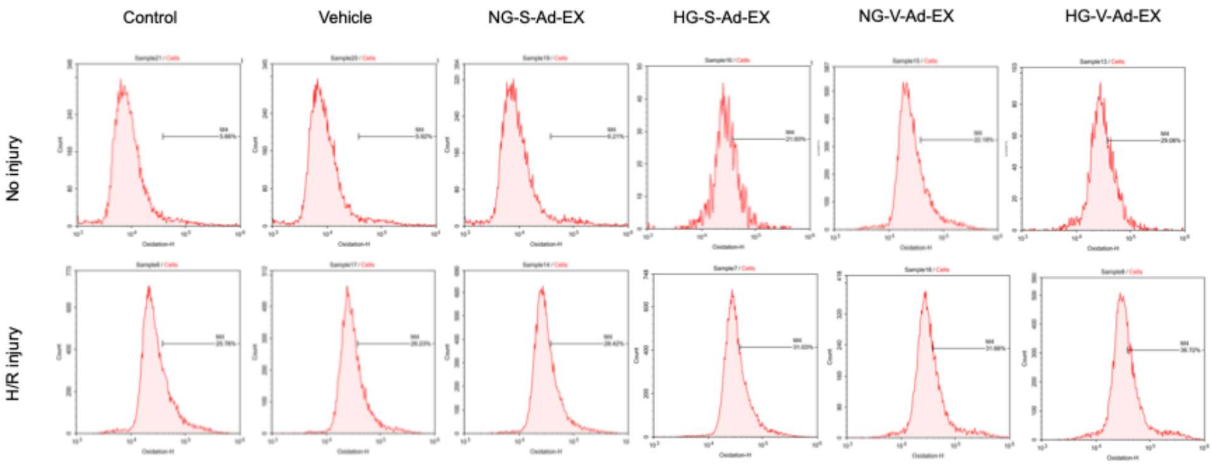

Supplemental Figure S3: HG-treated S-Ad-EXs and V-Ad-EXs increase the oxidative stress in HBMECs. Representative flow plots of dihydroethidium (DHE)-positive HBMECs.
